# Supplementary material for: Immune stimulation reduces sleep and memory ability in Drosophila melanogaster
Source: PeerJ. 2014 Jun 10;2:e434. doi: 10.7717/peerj.434 (PMC4060034; doi:10.7717/peerj.434)
Supplement: Supplemental Information [file peerj-02-434-s001.docx]

**Sleep bins**

Complete 4-way ANOVA

## Df Sum Sq Mean Sq F value Pr(>F)

## genotype 2 412877 206438 1170.68 < 2e-16 ***

## ru 1 23774 23774 134.82 < 2e-16 ***

## sex 1 5788 5788 32.82 1.0e-08 ***

## Time 71 5594618 78797 446.85 < 2e-16 ***

## genotype:ru 2 55984 27992 158.74 < 2e-16 ***

## genotype:sex 2 48468 24234 137.43 < 2e-16 ***

## ru:sex 1 4231 4231 23.99 9.7e-07 ***

## genotype:Time 142 231890 1633 9.26 < 2e-16 ***

## ru:Time 71 58074 818 4.64 < 2e-16 ***

## sex:Time 71 736868 10378 58.85 < 2e-16 ***

## genotype:ru:sex 2 7333 3666 20.79 9.5e-10 ***

## genotype:ru:Time 142 29910 211 1.19 0.057 .

## genotype:sex:Time 142 92833 654 3.71 < 2e-16 ***

## ru:sex:Time 71 77158 1087 6.16 < 2e-16 ***

## genotype:ru:sex:Time 142 44774 315 1.79 2.8e-08 ***

## Residuals 23976 4227952 176

## ---

## Signif. codes: 0 '***' 0.001 '**' 0.01 '*' 0.05 '.' 0.1 ' ' 1

4-way ANOVA for GS1.32>PGRP-Lca vs GS1.32/+

## Df Sum Sq Mean Sq F value Pr(>F)

## genotype 1 247192 247192 1387.82 < 2e-16 ***

## ru 1 8758 8758 49.17 2.4e-12 ***

## sex 1 20669 20669 116.04 < 2e-16 ***

## Time 71 4007124 56438 316.86 < 2e-16 ***

## genotype:ru 1 50295 50295 282.37 < 2e-16 ***

## genotype:sex 1 29880 29880 167.76 < 2e-16 ***

## ru:sex 1 5721 5721 32.12 1.5e-08 ***

## genotype:Time 71 93419 1316 7.39 < 2e-16 ***

## ru:Time 71 52509 740 4.15 < 2e-16 ***

## sex:Time 71 556096 7832 43.97 < 2e-16 ***

## genotype:ru:sex 1 5833 5833 32.75 1.1e-08 ***

## genotype:ru:Time 71 17248 243 1.36 0.023 *

## genotype:sex:Time 71 55887 787 4.42 < 2e-16 ***

## ru:sex:Time 71 62037 874 4.91 < 2e-16 ***

## genotype:ru:sex:Time 71 26367 371 2.09 2.6e-07 ***

## Residuals 16632 2962421 178

## ---

## Signif. codes: 0 '***' 0.001 '**' 0.01 '*' 0.05 '.' 0.1 ' ' 1

4-way ANOVA for GS1.32>PGRP-Lca vs +/ PGRP-Lca

## Df Sum Sq Mean Sq F value Pr(>F)

## genotype 1 14532 14532 88.65 < 2e-16 ***

## ru 1 67582 67582 412.26 < 2e-16 ***

## sex 1 13336 13336 81.35 < 2e-16 ***

## Time 71 3542271 49891 304.34 < 2e-16 ***

## genotype:ru 1 1938 1938 11.82 0.00059 ***

## genotype:sex 1 40695 40695 248.24 < 2e-16 ***

## ru:sex 1 7 7 0.04 0.83609

## genotype:Time 71 108904 1534 9.36 < 2e-16 ***

## ru:Time 71 31051 437 2.67 1.3e-12 ***

## sex:Time 71 369522 5205 31.75 < 2e-16 ***

## genotype:ru:sex 1 2 2 0.01 0.90374

## genotype:ru:Time 71 11016 155 0.95 0.60581

## genotype:sex:Time 71 32858 463 2.82 3.7e-14 ***

## ru:sex:Time 71 57250 806 4.92 < 2e-16 ***

## genotype:ru:sex:Time 71 7457 105 0.64 0.99197

## Residuals 15840 2596681 164

## ---

## Signif. codes: 0 '***' 0.001 '**' 0.01 '*' 0.05 '.' 0.1 ' ' 1

**Sleep bouts**

Complete 4-way ANOVA

## Df Sum Sq Mean Sq F value Pr(>F)

## genotype 2 163 81 78.75 < 2e-16 ***

## ru 1 6 6 5.65 0.01746 *

## sex 1 475 475 460.28 < 2e-16 ***

## Time 71 3854 54 52.58 < 2e-16 ***

## genotype:ru 2 144 72 69.90 < 2e-16 ***

## genotype:sex 2 59 29 28.40 4.8e-13 ***

## ru:sex 1 26 26 25.58 4.3e-07 ***

## genotype:Time 142 1406 10 9.59 < 2e-16 ***

## ru:Time 71 114 2 1.55 0.00204 **

## sex:Time 71 1052 15 14.35 < 2e-16 ***

## genotype:ru:sex 2 6 3 2.88 0.05597 .

## genotype:ru:Time 142 272 2 1.86 2.7e-09 ***

## genotype:sex:Time 142 787 6 5.37 < 2e-16 ***

## ru:sex:Time 71 120 2 1.64 0.00053 ***

## genotype:ru:sex:Time 142 214 2 1.46 0.00032 ***

## Residuals 23976 24751 1

## ---

## Signif. codes: 0 '***' 0.001 '**' 0.01 '*' 0.05 '.' 0.1 ' ' 1

4-way ANOVA for GS1.32>PGRP-Lca vs GS1.32/+

## Df Sum Sq Mean Sq F value Pr(>F)

## genotype 1 155 154.6 150.08 < 2e-16 ***

## ru 1 13 12.9 12.55 0.0004 ***

## sex 1 218 217.9 211.42 < 2e-16 ***

## Time 71 3194 45.0 43.66 < 2e-16 ***

## genotype:ru 1 13 13.5 13.08 0.0003 ***

## genotype:sex 1 3 3.3 3.24 0.0718 .

## ru:sex 1 17 17.4 16.84 4.1e-05 ***

## genotype:Time 71 711 10.0 9.72 < 2e-16 ***

## ru:Time 71 95 1.3 1.30 0.0475 *

## sex:Time 71 851 12.0 11.64 < 2e-16 ***

## genotype:ru:sex 1 6 5.9 5.71 0.0169 *

## genotype:ru:Time 71 159 2.2 2.18 4.0e-08 ***

## genotype:sex:Time 71 522 7.4 7.13 < 2e-16 ***

## ru:sex:Time 71 138 1.9 1.89 1.0e-05 ***

## genotype:ru:sex:Time 71 117 1.6 1.60 0.0010 **

## Residuals 16632 17138 1.0

## ---

## Signif. codes: 0 '***' 0.001 '**' 0.01 '*' 0.05 '.' 0.1 ' ' 1

4-way ANOVA for GS1.32>PGRP-Lca vs +/ PGRP-Lca

## Df Sum Sq Mean Sq F value Pr(>F)

## genotype 1 71 71 70.17 < 2e-16 ***

## ru 1 47 47 47.08 7.1e-12 ***

## sex 1 346 346 343.44 < 2e-16 ***

## Time 71 2645 37 36.95 < 2e-16 ***

## genotype:ru 1 72 72 71.52 < 2e-16 ***

## genotype:sex 1 55 55 54.60 1.6e-13 ***

## ru:sex 1 30 30 29.94 4.5e-08 ***

## genotype:Time 71 348 5 4.86 < 2e-16 ***

## ru:Time 71 163 2 2.28 5.7e-09 ***

## sex:Time 71 835 12 11.67 < 2e-16 ***

## genotype:ru:sex 1 1 1 0.80 0.3706

## genotype:ru:Time 71 108 2 1.51 0.0037 **

## genotype:sex:Time 71 338 5 4.72 < 2e-16 ***

## ru:sex:Time 71 133 2 1.86 1.6e-05 ***

## genotype:ru:sex:Time 71 94 1 1.32 0.0377 *

## Residuals 15840 15973 1

## ---

## Signif. codes: 0 '***' 0.001 '**' 0.01 '*' 0.05 '.' 0.1 ' ' 1

**Mean waking activity**

Complete 4-way ANOVA

## Df Sum Sq Mean Sq F value Pr(>F)

## genotype 2 450 225 87.03 < 2e-16 ***

## ru 1 91 91 35.05 3.3e-09 ***

## sex 1 1665 1665 644.21 < 2e-16 ***

## Time 71 4088 58 22.28 < 2e-16 ***

## genotype:ru 2 114 57 21.96 2.9e-10 ***

## genotype:sex 2 79 39 15.20 2.5e-07 ***

## ru:sex 1 332 332 128.27 < 2e-16 ***

## genotype:Time 142 1737 12 4.73 < 2e-16 ***

## ru:Time 71 219 3 1.19 0.1299

## sex:Time 71 764 11 4.17 < 2e-16 ***

## genotype:ru:sex 2 195 97 37.64 < 2e-16 ***

## genotype:ru:Time 142 732 5 1.99 2.4e-11 ***

## genotype:sex:Time 142 928 7 2.53 < 2e-16 ***

## ru:sex:Time 71 275 4 1.50 0.0042 **

## genotype:ru:sex:Time 142 427 3 1.16 0.0888 .

## Residuals 23976 61967 3

## ---

## Signif. codes: 0 '***' 0.001 '**' 0.01 '*' 0.05 '.' 0.1 ' ' 1

4-way ANOVA for GS1.32>PGRP-Lca vs GS1.32/+

## Df Sum Sq Mean Sq F value Pr(>F)

## genotype 1 217 217 90.60 < 2e-16 ***

## ru 1 12 12 4.98 0.0257 *

## sex 1 896 896 374.21 < 2e-16 ***

## Time 71 1781 25 10.48 < 2e-16 ***

## genotype:ru 1 1 1 0.49 0.4858

## genotype:sex 1 5 5 2.17 0.1404

## ru:sex 1 159 159 66.24 4.3e-16 ***

## genotype:Time 71 601 8 3.54 < 2e-16 ***

## ru:Time 71 141 2 0.83 0.8438

## sex:Time 71 760 11 4.47 < 2e-16 ***

## genotype:ru:sex 1 170 170 71.13 < 2e-16 ***

## genotype:ru:Time 71 487 7 2.86 1.4e-14 ***

## genotype:sex:Time 71 660 9 3.88 < 2e-16 ***

## ru:sex:Time 71 244 3 1.44 0.0094 **

## genotype:ru:sex:Time 71 184 3 1.08 0.3005

## Residuals 16632 39817 2

## ---

## Signif. codes: 0 '***' 0.001 '**' 0.01 '*' 0.05 '.' 0.1 ' ' 1

4-way ANOVA for GS1.32>PGRP-Lca vs +/ PGRP-Lca

## Df Sum Sq Mean Sq F value Pr(>F)

## genotype 1 41 41 15.46 8.5e-05 ***

## ru 1 98 98 37.27 1.1e-09 ***

## sex 1 1325 1325 502.04 < 2e-16 ***

## Time 71 4464 63 23.81 < 2e-16 ***

## genotype:ru 1 103 103 39.18 4.0e-10 ***

## genotype:sex 1 42 42 15.89 6.7e-05 ***

## ru:sex 1 523 523 197.98 < 2e-16 ***

## genotype:Time 71 583 8 3.11 < 2e-16 ***

## ru:Time 71 448 6 2.39 5.3e-10 ***

## sex:Time 71 863 12 4.60 < 2e-16 ***

## genotype:ru:sex 1 3 3 1.17 0.27941

## genotype:ru:Time 71 247 3 1.32 0.03801 *

## genotype:sex:Time 71 510 7 2.72 4.1e-13 ***

## ru:sex:Time 71 306 4 1.63 0.00065 ***

## genotype:ru:sex:Time 71 259 4 1.38 0.01900 *

## Residuals 15840 41817 3

## ---

## Signif. codes: 0 '***' 0.001 '**' 0.01 '*' 0.05 '.' 0.1 ' ' 1

**Sleep latency**

Complete 4-way ANOVA

## Df Sum Sq Mean Sq F value Pr(>F)

## genotype 2 18774 9387 13.13 2.4e-06 ***

## ru 1 4872 4872 6.81 0.00920 **

## sex 1 8532 8532 11.93 0.00058 ***

## Time 2 1958 979 1.37 0.25485

## genotype:ru 2 2545 1272 1.78 0.16935

## genotype:sex 2 33312 16656 23.29 1.4e-10 ***

## ru:sex 1 18731 18731 26.19 3.8e-07 ***

## genotype:Time 4 3636 909 1.27 0.27957

## ru:Time 2 6241 3121 4.36 0.01299 *

## sex:Time 2 1814 907 1.27 0.28176

## genotype:ru:sex 2 1001 500 0.70 0.49693

## genotype:ru:Time 4 9593 2398 3.35 0.00976 **

## genotype:sex:Time 4 6330 1583 2.21 0.06577 .

## ru:sex:Time 2 8663 4331 6.06 0.00244 **

## genotype:ru:sex:Time 4 18989 4747 6.64 2.9e-05 ***

## Residuals 925 661486 715

## ---

## Signif. codes: 0 '***' 0.001 '**' 0.01 '*' 0.05 '.' 0.1 ' ' 1

4-way ANOVA for GS1.32>PGRP-Lca vs GS1.32/+

## Df Sum Sq Mean Sq F value Pr(>F)

## genotype 1 11372 11372 27.54 2.1e-07 ***

## ru 1 1394 1394 3.38 0.0666 .

## sex 1 28113 28113 68.07 8.5e-16 ***

## Time 2 768 384 0.93 0.3952

## genotype:ru 1 947 947 2.29 0.1304

## genotype:sex 1 3157 3157 7.64 0.0059 **

## ru:sex 1 10583 10583 25.62 5.4e-07 ***

## genotype:Time 2 279 139 0.34 0.7138

## ru:Time 2 923 462 1.12 0.3277

## sex:Time 2 1435 718 1.74 0.1768

## genotype:ru:sex 1 63 63 0.15 0.6964

## genotype:ru:Time 2 554 277 0.67 0.5114

## genotype:sex:Time 2 1691 846 2.05 0.1298

## ru:sex:Time 2 912 456 1.10 0.3322

## genotype:ru:sex:Time 2 252 126 0.30 0.7373

## Residuals 663 273805 413

## ---

## Signif. codes: 0 '***' 0.001 '**' 0.01 '*' 0.05 '.' 0.1 ' ' 1

4-way ANOVA for GS1.32>PGRP-Lca vs +/ PGRP-Lca

## Df Sum Sq Mean Sq F value Pr(>F)

## genotype 1 15801 15801 18.46 2.0e-05 ***

## ru 1 2312 2312 2.70 0.10077

## sex 1 3439 3439 4.02 0.04544 *

## Time 2 2449 1225 1.43 0.23991

## genotype:ru 1 2486 2486 2.91 0.08879 .

## genotype:sex 1 32620 32620 38.12 1.2e-09 ***

## ru:sex 1 12649 12649 14.78 0.00013 ***

## genotype:Time 2 2228 1114 1.30 0.27277

## ru:Time 2 9065 4532 5.30 0.00525 **

## sex:Time 2 3263 1632 1.91 0.14947

## genotype:ru:sex 1 1029 1029 1.20 0.27320

## genotype:ru:Time 2 6365 3182 3.72 0.02482 *

## genotype:sex:Time 2 1831 916 1.07 0.34366

## ru:sex:Time 2 10439 5220 6.10 0.00239 **

## genotype:ru:sex:Time 2 16221 8111 9.48 8.9e-05 ***

## Residuals 600 513459 856

## ---

## Signif. codes: 0 '***' 0.001 '**' 0.01 '*' 0.05 '.' 0.1 ' ' 1

**Sleep bout duration**

Complete 4-way ANOVA

## Df Sum Sq Mean Sq F value Pr(>F)

## genotype 2 389225 194613 718.28 < 2e-16 ***

## ru 1 46608 46608 172.02 < 2e-16 ***

## sex 1 163298 163298 602.71 < 2e-16 ***

## Time 71 4004425 56400 208.16 < 2e-16 ***

## genotype:ru 2 110266 55133 203.49 < 2e-16 ***

## genotype:sex 2 44388 22194 81.91 < 2e-16 ***

## ru:sex 1 1998 1998 7.37 0.0066 **

## genotype:Time 142 125778 886 3.27 < 2e-16 ***

## ru:Time 71 61411 865 3.19 < 2e-16 ***

## sex:Time 71 542529 7641 28.20 < 2e-16 ***

## genotype:ru:sex 2 13490 6745 24.89 1.6e-11 ***

## genotype:ru:Time 142 49407 348 1.28 0.0129 *

## genotype:sex:Time 142 72259 509 1.88 1.4e-09 ***

## ru:sex:Time 71 57091 804 2.97 1.1e-15 ***

## genotype:ru:sex:Time 142 61540 433 1.60 8.0e-06 ***

## Residuals 23976 6496113 271

## ---

## Signif. codes: 0 '***' 0.001 '**' 0.01 '*' 0.05 '.' 0.1 ' ' 1

4-way ANOVA for GS1.32>PGRP-Lca vs GS1.32/+

## Df Sum Sq Mean Sq F value Pr(>F)

## genotype 1 129616 129616 514.26 < 2e-16 ***

## ru 1 7728 7728 30.66 3.1e-08 ***

## sex 1 144927 144927 575.01 < 2e-16 ***

## Time 71 2544070 35832 142.17 < 2e-16 ***

## genotype:ru 1 73213 73213 290.48 < 2e-16 ***

## genotype:sex 1 41224 41224 163.56 < 2e-16 ***

## ru:sex 1 4538 4538 18.00 2.2e-05 ***

## genotype:Time 71 53047 747 2.96 1.3e-15 ***

## ru:Time 71 38798 546 2.17 5.1e-08 ***

## sex:Time 71 404978 5704 22.63 < 2e-16 ***

## genotype:ru:sex 1 10479 10479 41.58 1.2e-10 ***

## genotype:ru:Time 71 30290 427 1.69 0.00025 ***

## genotype:sex:Time 71 32928 464 1.84 2.2e-05 ***

## ru:sex:Time 71 37051 522 2.07 3.4e-07 ***

## genotype:ru:sex:Time 71 35426 499 1.98 1.9e-06 ***

## Residuals 16632 4191966 252

## ---

## Signif. codes: 0 '***' 0.001 '**' 0.01 '*' 0.05 '.' 0.1 ' ' 1

4-way ANOVA for GS1.32>PGRP-Lca vs +/ PGRP-Lca

## Df Sum Sq Mean Sq F value Pr(>F)

## genotype 1 76200 76200 280.46 < 2e-16 ***

## ru 1 134162 134162 493.79 < 2e-16 ***

## sex 1 174214 174214 641.20 < 2e-16 ***

## Time 71 2958442 41668 153.36 < 2e-16 ***

## genotype:ru 1 2115 2115 7.78 0.0053 **

## genotype:sex 1 21121 21121 77.73 < 2e-16 ***

## ru:sex 1 1006 1006 3.70 0.0544 .

## genotype:Time 71 58753 828 3.05 < 2e-16 ***

## ru:Time 71 42064 592 2.18 4.0e-08 ***

## sex:Time 71 376198 5299 19.50 < 2e-16 ***

## genotype:ru:sex 1 0 0 0.00 0.9733

## genotype:ru:Time 71 17172 242 0.89 0.7332

## genotype:sex:Time 71 47693 672 2.47 9.5e-11 ***

## ru:sex:Time 71 52687 742 2.73 3.0e-13 ***

## genotype:ru:sex:Time 71 20638 291 1.07 0.3224

## Residuals 15840 4303741 272

## ---

## Signif. codes: 0 '***' 0.001 '**' 0.01 '*' 0.05 '.' 0.1 ' ' 1
